# The impact of successful chronic total occlusion percutaneous coronary intervention on clinical outcomes: a tertiary single-center analysis

**DOI:** 10.3389/fcvm.2024.1447829

**Published:** 2024-09-27

**Authors:** Maximilian Will, Konstantin Schwarz, Simone Aufhauser, Gregor Leibundgut, Elisabeth Schmidt, David Mayer, Paul Vock, Josip A. Borovac, Chun Shing Kwok, Gudrun Lamm, Julia Mascherbauer, Thomas Weiss

**Affiliations:** ^1^ Karl Landsteiner University of Health Sciences, Krems, Austria; ^2^ Division of Internal Medicine 3, University Hospital St. Pölten, St. Pölten, Austria; ^3^ Karl Landsteiner Institute for Cardiometabolics, Karl Landsteiner Society, St. Pölten, Austria; ^4^ Klinik für Kardiologie, Universitätsspital Basel, Basel, Switzerland; ^5^ Division of Interventional Cardiology, Cardiovascular Diseases Department, University Hospital of Split (KBC Split), Split, Croatia; ^6^ Department of Cardiology, Royal Stoke University Hospital, University Hospitals of North Midlands NHS Trust, Stoke-on-Trent, United Kingdom; ^7^ Medical School, Sigmund-Freud University, Vienna, Austria

**Keywords:** coronary artery disease (CAD), chronic total occlusion (CTO), percutaneous coronary intervention (PCI), quality of life, symptoms, mortality

## Abstract

**Background:**

The benefit of chronic total occlusion (CTO)-percutaneous coronary intervention (PCI) is controversial because of a lack of high-quality evidence. We aim to evaluate the impact of CTO-PCI on symptoms, quality of life and mortality.

**Methods:**

We conducted a retrospective single center study of patients with CTO-PCI in a tertiary center in Austria. The study outcomes were Canadian Cardiovascular Society (CCS) angina score, quality of life measured by Seattle Angina Questionnaire (SAQ), and death at median follow up for patients with successful vs. failed CTO-PCI.

**Results:**

A total of 300 patients underwent CTO-PCI for coronary artery disease, of which 252 (84%) were technically successful with median follow up of 3.4 years. There were no significant differences in in-hospital or all-cause mortality, major adverse cardiovascular event, or stent-related complications between the groups of failed and successful CTO-PCI. Among patients with successful CTO-PCI there was a significant improvement in CCS score, which was not found for the group with failed CTO-PCI. Successful reopening was associated with significant benefits of the SAQ domains of angina with stressful activity [3.7 ± 0.9 vs. 3.1 ± 0.5, *p* = 0.004, use of nitrates (4.7 ± 0.5 vs. 3.0 ± 1.0) *p* = 0.005] and satisfaction from angina relief (4.4 ± 1.1 vs. 3.6 ± 1.4 *p* < 0.001).

**Conclusion:**

While there was no significant difference in mortality, successful CTO-PCI was associated with greater reduction in angina and the use of nitrates compared to unsuccessful CTO-PCI.

## Introduction

Coronary angiography and percutaneous coronary intervention (PCI) are important cornerstones for the diagnosis and treatment of coronary artery disease (CAD) (1–3). The prevalence of coronary chronic total occlusions (CTO) in patients suffering from chronic coronary syndrome (CCS) is reported with 15%–25% (4, 5). Nevertheless, treatment of coronary CTOs remains uncommon in daily clinical practice (6, 7). Many cardiologists may hesitate to refer patients for revascularization due to the limited high-level evidence on its prognostic impact. Additionally, some operators may feel reluctant to attempt revascularization of CTO lesions because of procedural complexity, lack of specific skills, increased risks of complications, and the necessity for special equipment. However, recent strategical and technological advancements, improvement of equipment, better education, and training, as well as growing awareness of the high technical success (5, 8, 9) and low complication rates (8, 10) of CTO-PCI in the medical community, have lately resulted in the adoption of this treatment option in a larger population of patients (5, 11).

Despite the extensive research in the field of CTO-PCI over the last decades, whether chronic occlusions should be treated with optimal medical therapy (OMT) or PCI remains controversial. It is widely agreed that CTO-PCI has the potential to improve exercise limiting ischemic symptoms of angina or angina equivalents such as dyspnea in carefully selected patients (12–14). However, randomized controlled trials (RCT) have failed to demonstrate an effect of CTO-PCI on hard endpoints such as mortality (15). Widely, a major problem in this field of research is that CTO lesions are not well represented in large PCI trials. Generally, CTOs are excluded from RCTs. Specific RCTs on CTO-PCI often included less symptomatic patients since physicians may feel reluctant to randomize highly symptomatic patients to a conservative treatment arm. It is obvious that a difference in hard clinical endpoints in less symptomatic population at lower risk is difficult to demonstrate. Popular examples are the ORBITA (16) and ISCHEMIA (17) trials who failed to show a benefit for PCI over OMT in stable CAD. However, a recent, big observational study led by Park et al. (18) including 1,547 patients was able to show a long-term survival benefit of CTO-PCI. The primary endpoint, cardiac death at 10 years, was significantly reduced in the CTO-PCI cohort compared with the conservative treatment group. The findings of this study are consistent with previously reported observational data experiencing similar outcomes (19–21). However, to this day, no single large RCT on CTO-PCI vs. OMT has demonstrated a survival benefit of CTO recanalization.

The current state of CTO-PCI practice in Austria is largely unexplored. The aim of this project was to investigate if successful CTO-PCI leads to improvement in patient symptoms and prognosis in daily clinical practice.

## Methods

We retrospectively evaluated all consecutive CTO-PCI cases performed at a single tertiary care academic medical center in Austria from January 2016 until December 2021 that had a complete clinical follow-up. We compared the clinical, technical, and procedural characteristics, as well as patient-reported change in Canadian Cardiovascular Society (CCS) angina grade and Seattle Angina Questionnaire (SAQ). Furthermore, we examined all-cause mortality and major adverse cardiovascular events (MACE) between patients with technically successful vs. failed CTO-PCI procedures.

Coronary CTOs were defined as coronary lesions with Thrombolysis in Myocardial Infarction (TIMI) grade 0 flow of at least 3-month duration. Estimation of the duration of occlusion was clinical, based on the first onset of angina, prior history of MI in the target vessel territory, or comparison with a prior angiogram. Antegrade wire escalation (AWE) was defined as antegrade PCI during which the guidewire crossed the lesion from “true to true” lumen. A procedure was defined as retrograde if an attempt was made to cross the lesion through a collateral vessel or bypass graft supplying the target vessel distal to the lesion. Antegrade dissection/re-entry (ADR) was defined as antegrade PCI during which a guidewire was intentionally introduced into the subintimal space proximal to the lesion, or re-entry into the distal true lumen was attempted following intentional or inadvertent subintimal guidewire or device crossing.

Success was defined as successful CTO revascularization with achievement of <30% residual diameter stenosis within the treated segment and restoration of TIMI grade 3 antegrade flow. In-hospital MACE included any of the following adverse events prior to hospital discharge: all-cause mortality, recurrent symptoms requiring urgent repeat target vessel revascularization (TVR) with PCI or emergent coronary artery bypass graft (CABG) surgery and stroke.

Follow-up started from the date of the CTO procedure and ended on the first occurrence of either of: date of death, emigration, or end of the study (December 31, 2021). All patients who underwent CTO-PCI in the observation period were contacted and interviewed via phone to examine symptom improvement, if written informed consent was obtained.

Systolic function was evaluated by left ventricular ejection fraction (LVEF,%) measured by the Simpson’s 2D biplane method from transthoracic echocardiography.

## Statistical analysis

Statistical analysis was performed on Stata 13.0 (College Station, USA). Categorical variables were expressed as percentages and were compared using Pearson's Chi-square test or 2-tailed Fisher's exact test. Continuous variables were presented as mean ± standard deviation or median with interquartile range (IQR) and were compared using the *t*-test or Wilcoxon rank-sum test, as appropriate. For the comparison of the CCS class at baseline to follow up for successful and failed CTO-PCI, we used a paired *t*-test and changes in CCS class were presented in a figure. A Kaplan-Meier survival curve was constructed which was stratified by successful or failed CTO-PCI. Stepwise logistic regression with a *p*-value cutoff of 0.1 was used to identify factors associated with death and successful CTO-PCI. A two-sided *P*-value of 0.05 was considered statistically significant.

The Institutional Ethics Review Board of Karl Landsteiner University approved this study (Ethics Committee approval EK Nr: 1017/2021. The reporting of this study is in accordance with the STrengthening the Reporting of OBservational studies in Epidemiology (STROBE) recommendations (22). All procedures were undertaken in accord with Helsinki Declaration and postulates of good clinical practice.

## Results

In this retrospective analysis, we examined the clinical characteristics and procedural outcomes of 300 patients who underwent coronary angiography and percutaneous coronary intervention (PCI) of chronic coronary syndrome. We report that 48 CTO-PCI procedures were not successful (16%) while CTO-PCI was successful in 252 of patients (84%) according to predefined procedural success criteria.

The demographics and comorbidities of patients with CTO-PCI are shown in Table 1. The mean age of the entire cohort was 66.2 ± 9.9 years, with no statistically significant difference regarding age observed between the failed and successful CTO-PCI (67.0 ± 9.1 vs. 64.4 ± 10.3 years respectively, *p* = 0.11). Gender distribution showed a slight predominance of males. In terms of comorbidities, there were significantly fewer patients with regular alcohol consumption (44.6% vs. 60.4%, *p* = 0.045) and peripheral artery disease (9.9% vs. 29.2%, *p* < 0.001) in the group with successful CTO-PCI.

**Table 1:** Demographics and comorbidities of patients who underwent CTO-PCI.

Variable	Failed CTO-PCI (*N* = 48)		Successful CTO-PCI (*N* = 252)		*p*-value
Mean age (±SD)	67.0	±9.1	64.4	±10.3	0.11
	*N*	%	*N*	%	
Female	10	20.8	40	15.9	0.40
Smoking	26	54.2	133	52.8	0.86
Alcohol consumption	29	60.4	112	44.6	0.045*
Hypertension	36	75.0	186	74.1	0.90
Hyperlipidaemia	36	75.0	210	83.3	0.17
Familial hypercholesterolaemia	0	0.0	3	1.2	0.45
Diabetes mellitus	16	33.3	84	33.3	1.00
Diabetes status					1.00
Diet or no diabetes	33	68.8	173	69.2	
Insulin	4	8.3	21	8.4	
Oral	11	22.9	56	22.4	
Previous myocardial infarction	22	45.8	124	49.2	0.67
Previous PCI	39	81.3	209	82.9	0.78
Previous CABG	7	14.6	27	10.7	0.44
Peripheral artery disease	14	29.2	25	9.9	<0.001*
Cerebrovascular disease	8	16.7	32	12.7	0.46
Previous stroke or TIA	4	8.3	13	5.2	0.38
COPD	9	18.8	27	10.7	0.12
Malignancy	9	18.8	35	13.9	0.38
Atrial fibrillation	5	10.4	31	12.3	0.71
Heart failure	17	35.4	74	30.1	0.47

SD, standard deviation; PCI, percutaneous coronary intervention; CABG, coronary artery bypass graft; TIA, transient ischemic attack; COPD, chronic obstructive pulmonary disease. *p < 0.05.

The angina class and coronary lesion characteristics are shown in Table 2. There was no significant difference in CCS score for patients with successful and failed CTO-PCI. The proportion of patients with three-vessel coronary disease was significantly higher in patients with failed CTO-PCI compared to those with successful CTO-PCI (58.3% vs. 46.8%, *p* = 0.018, respectively). Among patients with successful CTO-PCI the mean number of stents was significantly greater (3.7 vs. 0.8, *p* < 0.001). No statistically significant differences were observed between two groups concerning in the utilization of rotablation.

**Table 2:** Angina class and lesion characteristics of patients who underwent CTO PCI.

Variable	Failed CTO-PCI (*n* = 48)		Successful CTO-PCI (*n* = 252)		*p*-value
	*N*	%	*N*	%	
CCS score					0.87
No angina	18	37.5	86	34.3	
1	2	4.2	22	8.8	
2	8	16.7	43	17.1	
3	9	18.8	44	17.5	
4	11	22.9	56	22.3	
No. of diseased vessels					0.018*
Single-vessel	9	18.8	48	19.1	
Two-vessel	10	20.8	83	32.9	
Three-vessel	18	37.5	103	40.9	
Single-vessel and left main	1	2.1	2	0.8	
Two-vessel and left main	0	0.0	1	0.4	
Three-vessel and left main	10	20.8	15	6.0	
Antegrade wiring	43	89.6	225	89.3	0.95
Rotablation	2	4.2	14	5.6	0.68
Mean no. of implanted stents (±SD)	0.8	±1.7	3.7	±2.2	<0.001*
Access site					ns
Right radial artery	8	17.8	37	14.9	
Left radial artery	2	4.4	21	8.5	
Right femoral artery	30	66.7	168	67.7	
Left femoral artery	2	4.4	5	2.0	
Left brachial artery	0	0.0	4	1.6	
Right femoral vein	2	4.4	12	4.8	
Left femoral vein	1	2.2	1	0.4	

SD, standard deviation. *p < 0.05.

The medications that were administered during the procedure are reported in Table 3. Periprocedural administration of most medications did not significantly differ between the two groups. There were modest differences in use of bivalirudin (*p* = 0.022), heparin (*p* = 0.001), nitroglycerine (*p* < 0.001), protamine sulfate (*p* < 0.001) and norepinephrine (*p* = 0.008). We observed significant differences in the use of dual antiplatelet therapy (DAPT) and duration of DAPT comparing the groups with successful and failed CTO-PCI.

**Table 3:** Medications of patients who underwent CTO-PCI.

Variable	Failed CTO-PCI (*n* = 48)		Successful CTO-PCI (*n* = 252)		*p*-value
	*N*	%	*N*	%	
Medications					
Adenosine	1	2.1	2	0.8	0.41
Aspirin	5	10.4	13	5.2	0.16
Atropine	1	2.1	6	2.4	0.90
Bivalirudin	1	2.1	0	0	0.022*
Ticagrelor	3	6.3	24	9.5	0.47
Piritramide	0	0.0	1	0.4	0.66
Urapidil	0	0.0	7	2.8	0.24
Prasugrel	0	0.0	7	2.8	0.24
Fentanyl	0	0.0	1	0.4	0.66
GP IIb/IIIa inhibitor	3	6.3	4	1.6	0.050
Isoptin	2	4.2	33	13.1	0.077
Furosemide	1	2.1	3	1.2	0.62
Nitroglycerine	9	18.8	138	54.8	<0.001*
Nitropohl infusion pump	1	2.1	1	0.4	0.19
Clopidogrel	7	14.6	41	16.3	0.77
Propofol	5	10.4	13	5.2	0.16
Protamine sulfate	2	4.2	0	0	<0.001*
PSP/Valium	2	4.2	3	1.2	0.14
Sedacorone	1	2.1	2	0.8	0.41
Epinephrine	1	2.1	3	1.2	0.62
Morphine	15	31.3	4	1.6	0.49*
Norepinephrine	4	8.3	0	0.0	0.008*
Mean heart rate (±SD)	69.4	±11.4	69.2	±12.2	0.90
Alpha-blocker	1	2.1	11	4.4	0.46
Beta-blocker	40	83.3	219	86.9	0.51
Calcium channel blocker	9	18.8	43	17.1	0.78
ACEi or ARB	42	87.5	203	80.6	0.25
Diuretic	20	41.7	93	36.9	0.53
Entresto	0	0	4	1.6	0.38
Aldosterone antagonist	4	8.3	39	15.5	0.20
Proton pump inhibitor	33	68.8	137	54.4	0.065
Statin					0.29
None	5	10.4	30	11.9	
Low/normal dose	20	41.7	76	30.2	
High dose	23	47.9	146	57.9	
Sedacoron	1	2.1	6	2.4	0.90
Antianginal drug	23	47.9	72	28.6	0.008*
Aspirin	46	95.8	250	99.2	0.062
DAPT medication					<0.001*
Clopidogrel	25	52.1	161	64.1	
Prasugrel	6	12.5	20	8.0	
Ticagrelor	10	20.8	68	27.1	
Other	7	14.6	2	0.8	
DAPT duration					<0.001*
1 month	0	0	1	0.4	
3 months	3	6.3	4	1.6	
6 months	3	6.3	12	4.8	
12 months	26	54.2	223	88.5	
24 months	2	4.2	3	1.2	
Other	14	29.0	9	3.5	
Triple therapy	5	10.4	30	12.0	0.76
Oral anticoagulant	6	12.5	34	13.5	0.85
Type of oral anticoagulant					
No anticoagulant	42	87.5	219	86.9	
Apixaban	1	2.1	8	3.2	
Dabigatran	0	0	2	0.8	
Edoxaban	0	0	1	0.4	
Rivaroxaban	3	6.3	14	5.6	
VKA	2	4.2	8	3.2	

SD, standard deviation; DAPT, dual antiplatelet therapy; VKA, vitamin K antagonist. *p < 0.05.

Echocardiographic and biochemical/laboratory characteristics of enrolled patients are shown in Supplementary Tables 1, 2, respectively. There were no significant differences in echocardiographic parameters between patients with successful and failed CTO-PCI. There were no differences in blood parameters for patients with successful vs. failed CTO-PCI aside from significantly greater pre-procedural hemoglobin count (13.9 vs. 13.3 g/dl, *p* = 0.031) and LDL cholesterol levels (93 vs. 80 mg/dl, *p* = 0.040).

**Figure 1:**
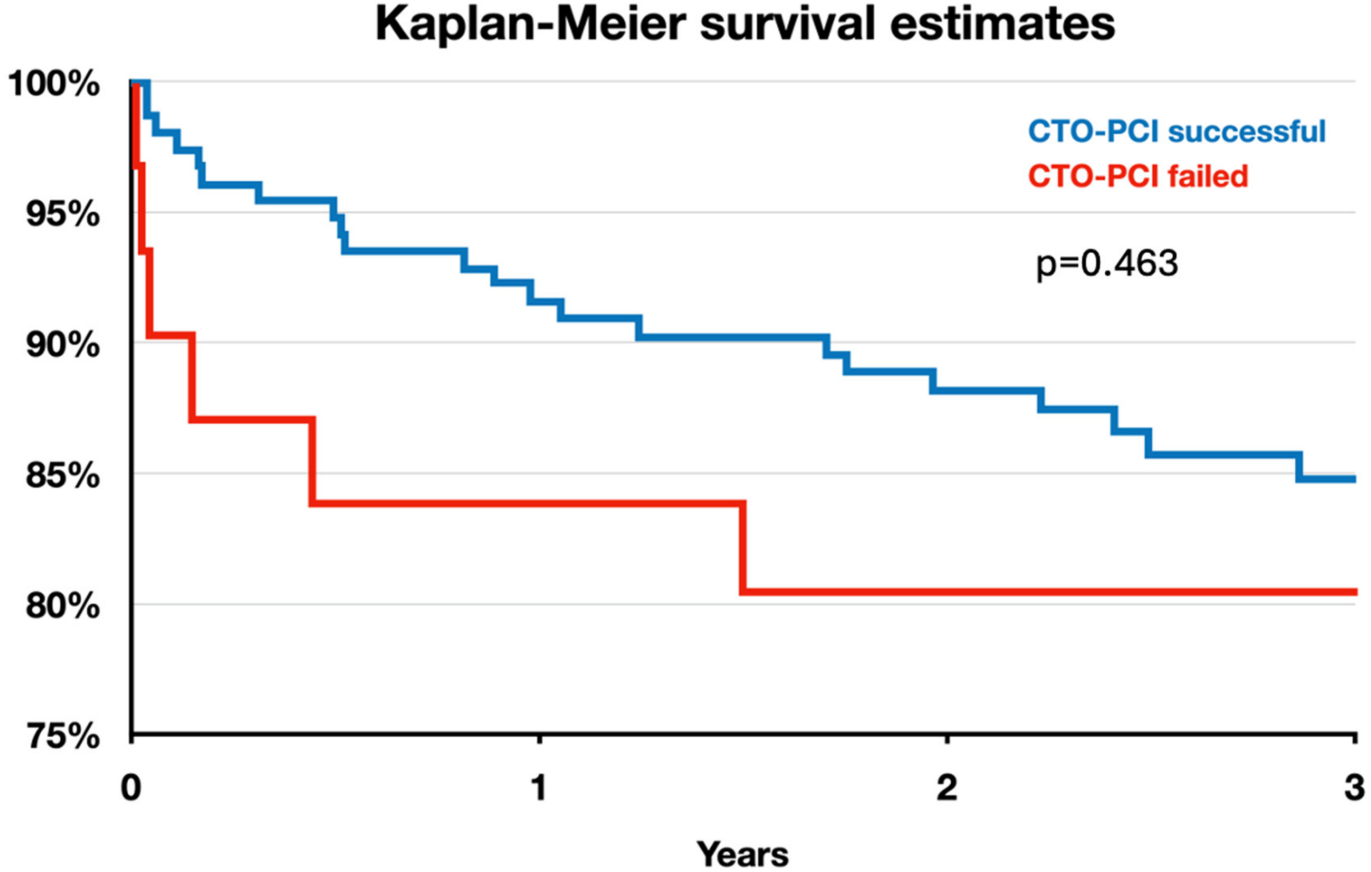
Kaplan meier survival curve for patients stratified by successful CTO-PCI.

Clinical outcomes of interest are shown in Table 4. Figure 1 shows the Kaplan-Meier survival curve for patients with successful and failed CTO-PCI for the first 3-years of follow-up. Overall mortality was 9.9% and 12.5%, respectively (*p* = 0.59)). There were no significant differences observed with respect to in-hospital death, or MACE between the two groups (3.6% vs. 2.1%, *p* = 0.60, and 8.7% vs. 6.3%, *p* = 0.57, respectively). Additionally, the incidence of in-stent thrombosis, its subtypes, and associated complications did not differ significantly between the two groups.

**Table 4:** Outcomes of patients who underwent CTO-PCI.

Variable	Failed CTO-PCI (*n* = 48)		Successful CTO-PCI (*n* = 252)		*p*-value
Mean follow up (±SD), days	1,152	±479	1,328	±512	0.12
	*N*	%	*N*	%	
Stent thrombosis	0	0	10	4.0	0.16
Stent thrombosis type					0.41
None	48	100	243	96.4	
Probable	0	0	1	0.4	
Definite	0	0	8	3.2	
Stent thrombosis time					0.62
None	48	100	243	96.4	
Acute	0	0	3	1.2	
Late	0	0	4	1.6	
Very late	0	0	2	0.8	
In-hospital death	1	2.1	9	3.6	0.60
All-cause death	6	12.5	25	9.9	0.59
MACE	3	6.3	22	8.7	0.57
MACE type					0.36
No MACE	45	93.8	230	91.3	
Cardiac death	0	0	6	2.4	
Myocardial infarction	3	6.3	7	2.8	
Revascularization	0	0	6	2.4	
Unknown	0	0	3	1.2	
Post intervention CCS class					0.20
No angina	11	45.8	72	56.7	
1	4	16.7	23	18.1	
2	3	12.5	16	12.6	
3	2	8.3	11	8.7	
4	4	16.7	5	3.9	

SD, standard deviation; MACE, major adverse cardiovascular event.

Post-procedural assessments included a comprehensive evaluation of patient-reported outcomes and QoL measures (Table 5). A total of 177 patients provided informed consent and completed the questionnaire via telephone interview. The successful CTO-PCI group exhibited favorable outcomes in terms of angina relief and overall satisfaction. More specifically, CTO-PCI was associated with a significant improvement in several SAQ domains, as follows: angina with stressful activity (*p* = 0.005), angina frequency in the last 4 weeks (*p* = 0.004), use of nitrates (*p* = 0.008) and satisfaction of no anginal relief (*p* = 0.002).

**Table 5:** SAQ at follow up.

Variable	Failed CTO-PCI (*n* = 48)		Successful CTO-PCI (*n* = 252)		*p*-value
SAQ-quality of Life^a^	*N*	±SD	*N*	±SD	
Getting dressed	4.8	±0.6	4.8	±0.6	0.68
Walking on flat surface	4.8	±0.4	4.8	±0.6	0.55
Showering	4.9	±0.4	4.7	±0.8	0.14
Walking uphill/stairs	4.0	±1.4	4.1	±1.6	0.74
Gardening/carrying shopping bags	4.2	±1.1	4.3	±1.2	0.77
Fast walking 100 m	3.9	±1.3	4.1	±1.5	0.57
Jogging/running	2.5	±1.8	3.9	±1.6	0.053†
Lifting heavy things	4.0	±1.5	4.3	±1.3	0.52
Strenuous exercise	3.9	±1.6	4.2	±1.5	0.60
Angina with stressful activity	3.1	±0.5	3.7	±0.9	0.004*
Angina frequency in last 4 weeks	3.2	±1.1	3.8	±1.2	0.091†
Use of nitrates	3.0	±1.0	4.7	±0.5	0.005*
Medication burden	4.3	±1.0	4.4	±1.1	0.87
Whether patient is convinced everything done for chest pain	4.5	±0.8	4.7	±0.7	0.18
Explanation by doctors satisfactory	4.6	±0.8	4.7	±0.8	0.58
Treatment success/satisfaction	4.5	±0.8	4.7	±0.8	0.21
Quality of life	4.3	±1.1	4.6	±1.0	0.22
Satisfaction of angina relief	3.6	±1.4	4.4	±1.1	<0.001*
How often they worry if they could have heart attack	4.0	±1.1	4.2	±1.0	0.25

SD, standard deviation; SAQ, seattle angina questionnaire.

^a^ Paired *t*-test.

* Significant (*p* = 0.05).

^†^ Trend (*p* < 0.10).

**Figure 2:**
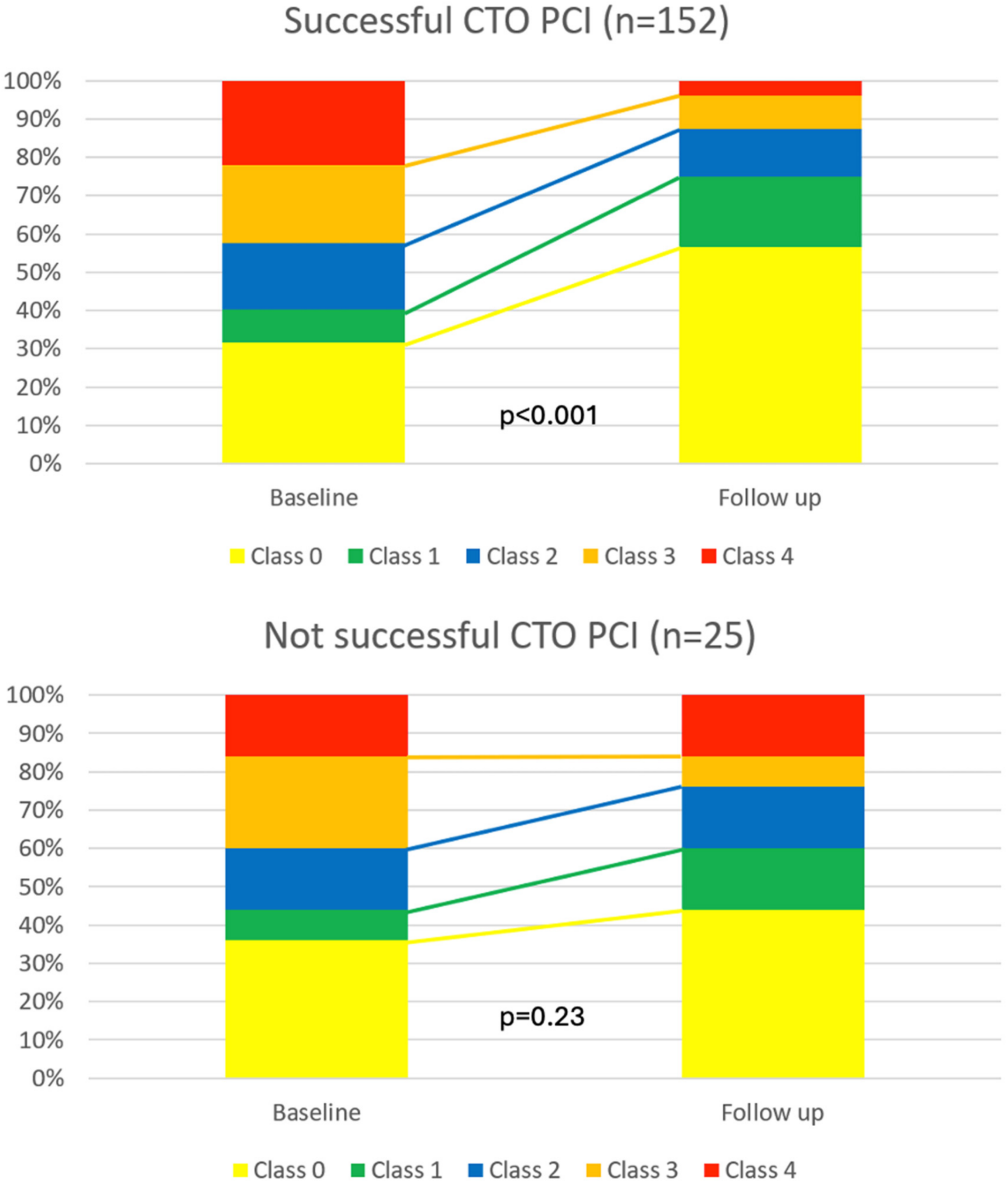
Change in CCS angina grade before and after follow-up for patients with chronic coronary syndrome and with successful vs. failed CTO-PCI.

The change in CCS class for patients with successful CTO-PCI and failed CTO-PCI is shown in Figure 2. Patients with successful CTO-PCI exhibited a significant improvement in follow-up vs. baseline self-reported CCS angina grade (*p* < 0.001). On the other hand, there was no statistical difference in CCS angina grade at follow-up vs. baseline in the group of patients that had failed CTO-PCI (*p* = 0.23).

Femoral access (OR 6.67, 95%-CI 1.69–26.38, *p* = 0.007), peripheral artery disease (PAD) (OR 2.95, 95%-CI 1.14–7.59, *p* = 0.025), heart failure (OR 2.37, 95%CI 1.06–5.31, *p* = 0.036), number of diseased vessels (OR 2.20, 95%-CI 1.15–4.21, *p* = 0.017) and heart rate (OR 1.05, 95%-CI 1.02–1.09, *p* = 0.002) were independently associated with higher mortality. The presence of PAD was the only independent predictor for failing CTO-PCI (OR 0.30, 95%-CI 0.14–0.66, *p* = 0.003). However, unsuccessful CTO-PCI was not independently associated with higher mortality.

## Discussion

The key finding of our study was that successful CTO-PCI significantly improved symptoms and quality of life during follow-up, among patients with chronic coronary syndrome.

Notably, one third of the investigated collective did not experience typical angina prior to undergoing PCI. Consequently, these patients were scheduled for revascularization to reduce ischemia in CCS (3) or to facilitate complete revascularization after ACS (23–25). This observed proportion is similar to previously published data from the OPEN-CTO by Sapontis and coworkers (26), where 72% underwent PCI with the primary indication to relief ischemic symptoms. Nevertheless, the proportion of patients with no angina at baseline was below 10% (26). However, some CTO patients may underestimate their daily symptom burden and perceive a significant improvement in quality of life after successful PCI that they may not have anticipated previously.

Widely, observational research and RCTs have provided substantial evidence indicating successful CTO-PCI is associated with improved angina relief and quality of life (27–29). Likewise, our study could demonstrate significantly improved angina measured by CCS following successful PCI in contradiction to failed PCI. Moreover, all but one parameter of SAQ were numerically better in patients with successful PCI compared to those with failed PCI in our study, In case of four variables (angina with stressful activity (*p* = 0.005), angina frequency in the last 4 weeks (*p* = 0.004), use of nitrates (*p* = 0.008) and satisfaction of no anginal relief (*p* = 0.002)) this difference reached statistical significance.

To this date, two randomized trials were published which compared the benefit of CTO-PCI vs. OMT in patients with stable angina (15, 28). The DECISION-CTO trial (15) enrolled 834 patients with stable angina including a CTO as one of their lesions. The results showed no difference between PCI vs. OMT regarding the primary endpoint of death, MI, stroke, or re-revascularization. Moreover, this trial also failed to show a significant difference for the secondary endpoint of symptomatic improvement measured by changes in the Seattle Angina Questionnaire (SAQ). However, several limitations need to be considered when interpreting the results of this trial. Most importantly, the study design predetermined that non-CTO lesions were treated after the baseline assessment in both groups. Given the fact that 77% of patients in DECISION-CTO have multi-vessel disease, around 70% of patients in the conservative arm received PCI, which could possibly explain the improvement in SAQ in the conservative arm. Furthermore, there was a high cross-over rate of about 20% from the conservative arm to the CTO-PCI group. Hence, when the results were reported in as per-treated analyses (rather than as per-protocol), there was a significant decrease in MACE and spontaneous MI (both *p* = 0.01), a strong trend to reduced all-cause mortality (*p* = 0.06) and cardiac mortality (*p* = 0.08) in the CTO-PCI treated group when compared to conservative treatment. In the EURO-CTO trial (28) with 448 randomized patients, CTO-PCI outperformed OMT in terms of symptomatic benefit. Contrary to DECISION-CTO, all patients in this study were randomized after treatment of concomitant relevant non-CTO lesions. Consistently, confounding effects of non-CTO-PCI were negligible in this study since a rather low cross-over rate of 7% from OMT in the intention-to-treat analysis was reported. The results showed significant differences in improvement in angina frequency and quality of life in SAQ subscales favoring CTO-PCI. Regarding major adverse cardiovascular and cerebrovascular events, no significant differences between the two groups were observed. Although the results of both studies may seem contradictory at first glance, DECISION-CTO does not disprove the results of the EURO-CTO study, because of the major differences in study design and the given limitations. Whereas DECISION-CTO failed to proof the benefit of CTO-PCI on top of Non-CTO-PCI, EURO-CTO identified the isolated benefit of CTO-PCI vs. OMT. Recently, at 3 years follow-up of the EURO-CTO trial observed no difference in the incidence of cardiovascular mortality or myocardial infarction between PCI or OMT. However, a higher MACE rate was observed in the OMT group, which was primarily due to ischemia driven revascularization (9).

Several research on the population of patients with CAD have emphasized the significant influence of CTOs on outcomes (18, 21, 30–33). Several underlying mechanisms have been reported for this phenomenon. Patients with CTOs may be prone to experience fatal cardiac events in the future. Notably, patients with STEMI who receive primary PCI experience a threefold rise in 30-day mortality if there is a bystander CTO in a non-culprit artery (21).

Occasionally, the culprit vessel in acute STEMI serves as donor artery for a myocardial CTO territory by providing collateral supply. In these cases of “double jeopardy” mortality increases up to 52%, because of a large area of threatened myocardium and a higher risk for cardiogenic shock (21).

This is attributed to the “double jeopardy” phenomenon, which occurs when the sudden blockage of a donor vessel supplying collateral blood flow beyond the CTO poses a threat to a larger myocardial area (21, 34). Additionally, non-revascularized CTOs may be associated with impaired left ventricular ejection fraction (LVEF), a well-established prognostic marker for MACE (4). Malignant arrhythmias also appear to play a significant role in causing cardiovascular fatalities among patients with CTOs (30, 35, 36).

Despite all these pathophysiological considerations, our study did not find a benefit of successful CTO-PCI on hard clinical endpoints. There was an absolute decrease in mortality by 2.6%, favoring successful CTO-PCI (9.9% vs. 12.5%), but likely due to small sample size this numerical difference did not reach statistical significance. In the event of a subtle effect size a rather small study like ours might fail to discern it. Moreover, the mean follow-up time of 3.4 years of our study may be too short to detect a significant difference in all-cause death since data by Park et al. (18) suggest later benefits of CTO revascularization.

Comparable to our study, Lee et al. (37) and Yamamoto et al. (38) conducted studies that found no significant difference in the survival rates of patients who underwent successful CTO-PCI at 3-year and 4.6-year follow-ups, respectively. However, both trials reported a TVR rate of 20% after failed CTO-PCI, suggesting that a considerable number of patients initially labeled as having an “unsuccessful” procedure were successfully revascularized later. However, meta-analyses have shown that successful CTO-PCI was associated with a lower risk of MACE and death compared with failed procedures (39, 40). Christakopoulos et al. (40) analyzed 25 observational studies and found successful CTO-PCI was associated with lower risk of death [odds ratio (OR), 0.52; 95% CI, 0.43–0.63] and MACE (OR 0.59; 95% CI, 0.44–0.79), but not TVR (OR, 0.66; 95% CI, 0.36–1.23) or MI (OR, 0.73; 95% CI, 0.52–1.03) compared with failed CTO-PCI, during a median follow-up of 3 years. In the report by Megaly et al. (39), which represents a contemporary CTO-PCI population since 2010, the risk of MACE and death was significantly lower at 12 months in patients who had successful CTO-PCI.

In conclusion, our analysis demonstrated a symptomatic benefit of successful CTO-PCI among patients with chronic coronary syndrome in a nationally representative tertiary medical center in Austria. Further research, including larger observational studies and randomized controlled trials, are necessary to investigate the effects of CTO-PCI on symptom relief and mortality rates.

### Limitations

Our study has some limitations worth discussing. First, this is a retrospective single center experience.

Second, this study represents information obtained from PCIs carried out from 2016 to 2021. However, this data may be considered outdated as it does not reflect the current state of the art in CTO-PCI due to technological advancements and improvements that occurred in this field over the last several years. Consequently, any impact on mortality and symptom improvements may have been underestimated, however, this notion should be investigated in future studies.

Another limitation is the extremely limited sample size of the unsuccessful group of only 25 probands, which is inadequate for making definitive conclusions. Notably, the proportion of patients with three-vessel coronary disease was significantly higher in patients with failed CTO-PCI compared to those with successful CTO-PCI which might be a major confounder in this setting. Furthermore, it cannot be excluded that the improvement of symptoms may have been attributable to the revascularization of non-chronically occluded coronary arteries rather than the chronic total occlusion lesion itself in these patients.

Moreover, one-third of the patients did not report typical angina, which was the primary endpoint of the study, prior to receiving PCI. However, this observation becomes even more intriguing considering that a significant difference was observed for this outcome. Regrettably, baseline values for the SAQ - scores pre-PCI were not available, limiting our ability to compare post PCI values and thus reducing the significance of this analyzed parameter.

One further constraint of our study is the absence of detailed information about ischemia testing for all patients, restricting a thorough analysis concerning this important aspect.

This analysis was limited to patients who underwent percutaneous coronary intervention, excluding those initially managed with optimal medical therapy alone. Consequently, the ability to compare outcomes between optimal medical management and successful PCI was constrained, potentially introducing selection bias and neglecting the placebo effect.

## Conclusions

Successful CTO-PCI significantly improved the symptoms of angina and quality of life during follow-up, among patients with chronic coronary syndrome. CTO-PCI should be considered in symptomatic patients alongside OMT. Prospective studies with larger sample size become are warranted to further address this important research question.

## Data Availability

The raw data supporting the conclusions of this article will be made available by the authors, without undue reservation.
