# Supplementary material for: The impact of successful chronic total occlusion percutaneous coronary intervention on clinical outcomes: a tertiary single-center analysis
Source: Front Cardiovasc Med. 2024 Sep 27;11:1447829. doi: 10.3389/fcvm.2024.1447829 (PMC11470476; doi:10.3389/fcvm.2024.1447829)
Supplement: Supplementary file 1 [file Table1.pdf]

Supplementary Table 1. Echocardiographic findings of patients who underwent CTO-PCI

**Variable**	**Failed CTO-PCI** (n=48)		**Successful CTO-PCI** (n=252)		**p-value**
	N	%	N	%	
LV function					0.55
Good function	27	56.3	139	57.0	
Slightly reduced	10	20.8	40	16.4	
Low-moderate reduction	2	4.2	8	3.2	
Moderate reduction	1	2.1	25	10.3	
Moderate to severe reduction	1	2.1	5	2.1	
Severe reduction	7	14.6	27	11.1	
LV configuration					0.26
Normal	38	79.2	208	85.6	
Dilated	10	20.8	35	14.4	
Diastolic impairment					0.060†
None	18	37.5	125	50.8	
Grade 1	22	45.8	76	30.9	
Grade 2	3	6.3	32	13.0	
Grade 3	5	10.4	13	5.3	
Left ventricular					0.77
hypertrophy	21	43.8	116	47.2	
None	20	41.7	96	39.0	
Mild	7	14.6	30	12.2	
Moderate	0	0.0	4	1.6	
Severe					
Dilated right ventricle	4	8.7	11	4.5	0.24
Right ventricular function					0.39
Normal	41	91.1	214	88.1	
Mildly reduced	0	0	13	5.4	
Moderately reduced	2	4.4	10	4.1	
Severely reduced	2	4.4	6	2.5	
Regional wall motion abnormality	28	58.3	129	52.4	0.45
Diffuse regional wall motion abnormality	4	8.3	27	11.0	0.59

Location of regional wall motion abnormality					
Basal anteriorseptal	4	8.3	14	5.7	0.49
Basal inferiorseptal	9	18.8	33	13.4	0.33
Basal inferior	21	43.8	85	34.6	0.23
Basal anterolateral	2	4.2	19	7.7	0.38
Mid anterior	3	6.3	10	4.1	0.50
Mid anteroseptal	2	4.2	18	7.3	0.43
Mid inferoseptal	6	12.5	21	8.5	0.38
Mid inferior	6	12.5	28	11.4	0.83
Mid anterolateral	2	4.2	13	5.3	0.75
Apical anterior	5	10.4	36	14.6	0.44
Apical septal	11	22.9	46	18.7	0.50
Apical inferior	12	25.0	40	16.3	0.15
Apical lateral	6	12.5	26	10.6	0.69
Posterolateral	18	37.5	59	24.0	0.051†
Aortic valve thickening	9	18.8	37	15.2	0.53
Aortic valve calcification	1	2.1	10	4.1	0.51
Aortic valve sclerosis	19	39.6	77	31.3	0.26
Aortic valve insufficiency	10	20.8	56	22.8	0.77
Mitral insufficiency	35	72.9	148	60.2	0.095†
Tricupsid insufficiency	19	39.6	72	29.3	0.16
Systolic pulmonary					0.21
pressure	11	29.0	72	43.9	
Not tested	21	55.3	67	40.9	
No increased	6	15.8	25	15.2	
Increased					
Pericardial effusion	1	2.1	7	2.9	0.77
Ascending aorta diameter					0.72
0	39	81.3	189	76.8	
1	7	14.6	48	19.5	
2	2	4.2	9	3.7	

Abbreviations: LV=left ventricular

Supplementary Table 2. Blood parameters and chronic kidney disease staging of patients who underwent CTO-PCI

**Variable**	**Failed CTO-PCI** (n=48)		**Successful CTO-PCI** (n=252)		**p-value**
**Pre-procedure**					
Hb	13.3	±1.8	13.9	±1.6	0.031*
Haematocrit	39.3	±5.3	40.8	±4.4	0.053†
Platelet	259	±123	240	±83	0.19
Leukocytes	7.9	±3.2	8.2	±3.3	0.56
CRP	0.6	±1.6	1.1	±2.7	0.26
Sodium	140	±5	141	±8	0.36
Potassium	4.5	±0.5	4.4	±0.5	0.16
Creatinine	1.1	±0.4	1.1	±0.7	0.67
eGFR	74	±33	70	±20	0.32
CKD stage<br>KDIGO1<br>KDIGO2<br>KDIGO3a<br>KDIGO3b<br>KDIGO4<br>KDIGO5	 5<br>29<br>5<br>3<br>1<br>0	 11.6%<br>67.4%<br>6.0%<br>6.0%<br>2.3%<br>0%	 28<br>146<br>27<br>10<br>2<br>2	 13.0%<br>67.9%<br>12.6%<br>4.7%<br>0.9%<br>0.9%	0.92
BUN	19.2	±10.4	19.1	±10.3	0.98
Glucose	130	±46	124	±50	0.50
CK	133	±132	175	±343	0.43
hsTroponin	68	±143	413	±2195	0.32
ProBNP	1454	±2611	953	±2010	0.39
NTproBNP	1262	±2273	1654	±3699	0.63
Cholesterol	142	±29	153	±44	0.15
HDL	50	±13	47	±14	0.26
LDL	80	±28	93	±37	0.040*
Triglycerides	130	±60	156	±98	0.12
HbA1c	6.1	±0.7	6.3	±0.9	0.29
**Post-procedure**					
Hb	12	±3	13	±2	0.18
Creatinine	1.1	±0.4	1.2	±0.9	0.49
eGFR	75	±39	70	±27	0.38
Post-procedure CKD<br>stage<br>KDIGO1<br>KDIGO2<br>KDIGO3a<br>KDIGO3b<br>KDIGO4<br>KDIGO5	 3<br>16<br>6<br>1<br>1<br>0	 11.1%<br>59.3%<br>22.2%<br>3.7%<br>3.7%<br>0%	 21<br>86<br>23<br>7<br>4<br>2	 14.7%<br>60.1%<br>16.1%<br>4.9%<br>2.8%<br>1.4%	0.94
CK	191	±446	167	±320	0.69
hs Troponin	308	±741	380	±1444	0.77

Abbreviations: **Hb**=hemoglobin, **CKD**=chronic kidney disease, **BUN**=blood urea nitrogen, **CK**=creatinine kinase, **eGFR**=estimated glomerular filtration rate, **HbA1c**=hemoglobin A1C, **BNP**=brain natriuretic peptide
